# Draft Genome Sequence of *Bacillus subtilis* Ia1a, a New Strain for Poly-γ-Glutamic Acid and Exopolysaccharide Production

**DOI:** 10.1128/genomeA.01361-16

**Published:** 2016-12-15

**Authors:** Mohammad H. A. Ibrahim, Brady F. Cress, Robert J. Linhardt, Mattheos A. G. Koffas, Richard A. Gross

**Affiliations:** a Department of Chemistry and Chemical Biology, Center for Biotechnology and Interdisciplinary Studies, Rensselaer Polytechnic Institute, Troy, New York, USA; b Department of Chemical and Biological Engineering, Rensselaer Polytechnic Institute, Troy, New York, USA; c Department of Biological Sciences, Rensselaer Polytechnic Institute, Troy, New York, USA; d Chemistry of Natural and Microbial Products Department, National Research Centre, Cairo, Egypt

## Abstract

We report here the 4.092-Mb high-quality draft genome assembly of a newly isolated poly-γ-glutamic acid–producing strain, *Bacillus subtilis* Ia1a. The genome sequence is considered a critical tool to facilitate the engineering of improved production strains. Exopolysaccharides and many industrially important enzymes can be produced by this new strain utilizing different carbon sources.

## GENOME ANNOUNCEMENT

The endospore-forming, rod-shaped aerobic species *Bacillus subtilis* and its relatives are well-characterized Gram-positive bacteria (1). Several *B. subtilis* strains have been isolated from soil and water sources, showing great ability to adapt to a wide spectrum of environments (2). Important industrial enzymes (protease, amylase, etc.) (3) and valuable metabolites (poly-γ-glutamic acid [γPGA], exopolysaccharides [EPSs], and antibiotics) are known to be produced by different strains of this species (4, 5).

Strain Ia1a was isolated from an Egyptian soil sample and is able to produce EPSs utilizing different carbon substrates such as glycerol, glucose, sucrose, molasses, and starch. In the presence of glutamic acid, high-volumetric production of large-molecular-size γPGA can be achieved by the strain after a relatively short cultivation time (unpublished data). Phylogenetic analysis of the 16S rRNA genes was used to identify strain Ia1a as a new strain of the species *B. subtilis*. Since the release of the first complete genome sequence of *B. subtilis* (6), comparative genomics of assorted *B. subtilis* strains has revealed several interesting metabolic pathways—such as those involved in sporulation and biofilm formation—that confer to *B. subtilis* a remarkable adaptation capability and that contribute to predominance in diverse environments (7).

For isolation of genomic DNA, a single colony of strain Ia1a grown on a medium E plate (8) was used to inoculate 50 mL of the same medium for shake-flask cultivation. The culture was grown at 37°C for 24 h, and harvested cells were then washed with distilled water and used for genomic DNA extraction and purification with a PureLink genomic DNA minikit (Invitrogen). The genomic DNA library was prepared using a Nextera DNA sample preparation kit (Illumina) following the manufacturer’s user guide. Subsequently, genomic DNA was subjected to simultaneous fragmentation and addition of adapter sequences. These adapters were utilized during a limited-cycle (five cycles) PCR, in which unique indices were added to the sample. Following library preparation, the average library size (750 bp) was determined using an Agilent 2100 bioanalyzer (Agilent Technologies). The library was diluted (to 12 pM), and paired-end reads were obtained using the MiSeq system for 600 cycles (Illumina).

The genome of strain Ia1a was assembled *de novo* by MR DNA (Shallowater, TX, USA) using NGen (DNASTAR, Inc.). Draft genome annotation was performed with the RAST server (9). The genome of strain Ia1a comprises 4,092,291 bp, possesses a GC content of 43.8%, and contains 4,201 coding sequences. The closest neighbor strains were *B. subtilis* 168, SC-8, and QB928, respectively. The complete genome sequence of strain Ia1a should facilitate metabolic understanding and subsequent engineering for designing improved strains for γPGA and EPS production. Moreover, it will enrich the available *B. subtilis* genome library and contribute to a better understanding of these industrially important organisms.

### Accession number(s).

The annotated genome sequence was deposited in DDBJ/EMBL/GenBank under accession number FMSN00000000. The version described in this paper is the first version, FMSN01000000.

## References

[1] Wipat A, Harwood CR 1999 The *Bacillus subtilis* genome sequence: the molecular blueprint of a soil bacterium. FEMS Microbiol Ecol 28:1–9. doi:10.1111/j.1574-6941.1999.tb00555.x.

[2] Earl AM, Losick R, Kolter R 2008 Ecology and genomics of *Bacillus subtilis*. Trends Microbiol 16:269–275. doi:10.1016/j.tim.2008.03.004. 18467096 PMC2819312

[3] Van Dijl JM, Hecker M 2013 *Bacillus subtilis*: from soil bacterium to super-secreting cell factory. Microb Cell Fact 12:3. doi:10.1186/1475-2859-12-3. 23311580 PMC3564730

[4] Schallmey M, Singh A, Ward OP 2004 Developments in the use of *Bacillus* species for industrial production. Can J Microbiol 50:1–17. doi:10.1139/w03-076. 15052317

[5] Marvasi M, Visscher PT, Casillas Martinez L 2010 Exopolymeric substances (EPS) from *Bacillus subtilis*: polymers and genes encoding their synthesis. FEMS Microbiol Lett 313:1–9. doi:10.1111/j.1574-6968.2010.02085.x. 20735481

[6] Kunst F, Ogasawara N, Moszer I, Albertini AM, Alloni G, Azevedo V, Bertero MG, Bessières P, Bolotin A, Borchert S, Borriss R, Boursier L, Brans A, Braun M, Brignell SC, Bron S, Brouillet S, Bruschi CV, Caldwell B, Capuano V, Carter NM, Choi SK, Codani JJ, Connerton IF, Danchin A 1997 The complete genome sequence of the Gram-positive bacterium *Bacillus subtilis*. Nature 390:249–256. doi:10.1038/36786. 9384377

[7] Vlamakis H, Chai Y, Beauregard P, Losick R, Kolter R 2013 Sticking together: building a biofilm the *Bacillus subtilis* way. Nat Rev Microbiol 11:157–168. doi:10.1038/nrmicro2960. 23353768 PMC3936787

[8] Leonard CG, Housewright RD, Thorne CB 1958 Effects of some metallic ions on glutamyl polypeptide synthesis by *Bacillus subtilis*. J Bacteriol 76:499–503. 13598708 10.1128/jb.76.5.499-503.1958 PMC290228

[9] Aziz RK, Bartels D, Best AA, DeJongh M, Disz T, Edwards RA, Formsma K, Gerdes S, Glass EM, Kubal M, Meyer F, Olsen GJ, Olson R, Osterman AL, Overbeek RA, McNeil LK, Paarmann D, Paczian T, Parrello B, Pusch GD, Reich C, Stevens R, Vassieva O, Vonstein V, Wilke A, Zagnitko O 2008 The RAST server: rapid annotations using subsystems technology. BMC Genomics 9:75. doi:10.1186/1471-2164-9-75. 18261238 PMC2265698

